# 

**Published:** 1825-09

**Authors:** 


### MONTHLY CATALOGUE OF MEDICAL BOOKS.

*** *It having been hinted to us that gentlemen, sending copies of their Works, prefer having their titles given at length, it is our intention, in future, to comply with this suggestion: but it is to be observed, that no books can be entered on this List except those sent to us for the purpose;—finding, in the lists hitherto transmitted, that the names of works have frequently been given as published, which have not appeared for weeks, or even months, after.*

Elements of the Theory and Practice of Physic; designed for the use of Students. By GEORGE GREGORY, M.D. Licentiate of the Royal College of Physicians in London: Secretary to the Medical and Chirurgical Society of London; Physician to the Small-Pox and Vaccination Hospital at St. Pancras; and senior Physician to the St. George's and St. James's Dispensary. Second Edition, with Additions and Amendments.—London, 1825.

Remarks on Irritative Fever, commonly called the Dock-yard Disease; with Mr. DRYDEN's detailed Account of the Fatal Cases, including that of the lamented Surgeon, Dr. BELL. Dedicated, with permission, to Commissioner SHIELD. By JOHN BUTTER, M.D. F.R.S. F.L.S. and S.W. &c. &c.—Devonport, 1825.

Practical Observations on certain Pathological Relations which exist between the Kidneys and other Organs of the Human Body, especially the Brain, Mucous Membranes, and Liver. By JOHN FOSBROKE, Surgeon, Cheltenham.—Cheltenham, 1825.

The Syphonic Theory; or, Brief Observations on the Circulation of the Blood, and on Respiration as connected therewith. By EDWARD HOPLEY, Surgeon, R.N. —London, 1825.

A Treatise on the Properties and Medicinal Application of the Vapour-Bath, in its different Varieties, and their Effects in various Species of Diseased Action. By J. GIBNEY, M.D. of the University of Edinburgh, Resident Physician at Brighton, &c. &c.—London, 1825.

A Synopsis, exhibiting at one view the probable Results, ex Ordine, of the various Chemical Processes contained in the Pharmacopœia Londinensis 1824. Together with Test Questions of the Pupil's Knowledge of the same.—London, 1825. Printed in one sheet.

### NOTICE TO CORRESPONDENTS.

*We have to acknowledge the receipt of* Dr. STOKER'S *Paper, through* Dr. J. JOHNSON.

*Communications have also been received from* Mr. ELLIOTT, Mr. HARTLE, Mr. MORLEY, Mr. HUTCHINSON, *of Southwell,* Mr. WALLER, *&c. &c.*

